# Errors of Refraction

**Published:** 1889-10

**Authors:** 


**Errors of Refraction.** By FRANCIS VALK, M. D., New York. New York and London: G. P. Putnam's Sons. 1889.

Under this title are collected and published the lectures delivered by the author to his class at the New York Post-Graduate School. This monograph of 241 pages is composed of eleven lectures, each being in itself a concise and complete exposition of the subject under discussion.

Lecture I. treats of the Anatomy of the Eye, and is accompanied by numerous well-selected illustrations, some of which are reproductions of blackboard sketches. The author describes the different anatomical parts of the eye in a simple adequate manner, enabling his hearers to become acquainted with the most important facts, without burdening them with the detail of close anatomical and histological research.

Describing the action of the ciliary muscle, the author refers to the description given in Flint's physiology as the best:

> When this muscle contracts, the choroid is drawn forward with, probably, a slightly spiral motion on the lens, the contents of the globe, situated posteriorly to the lens, are compressed, and the suspensory ligament is relaxed. The lens itself, the compressing and flattening action of the suspensory ligament being diminished, becomes thicker and more convexed, by virtue of its own elasticity.

Lecture II. treats of refraction, the power of the eye in bringing rays of light to a focus on the retina, and the image there produced. The discussion of the laws of optics, and their application to the human eye, the refracting power of the various lenses and the explanation of the old and new methods of notation are very ably set forth in this chapter. For a more careful study of this chapter, the writer refers his hearers to Landolt's "Refraction and Accommodation."

In Chapters III., IV. and V. the writer takes up Emmetropia, Hypermetropia and Myopia. Each of these states of refraction are illustrated by imaginary cases, testing for vision with Snellen's letters, and correcting the same by means of proper glasses.

His opinions as to the cause of Myopia, about which so much difference of opinion exists, are summed up in the following words:

> Myopia is either congenital or acquired, as the case may be, though I believe that there are very few persons born myopic, except those who may inherit it. While in the acquired form, as you will find in many students, I think it is due to the constant straining and congestion of the eye, produced by the dependent position of the head when a person is leaning forward to read; also, from the constant pressure that is exerted upon the sides of the globe by the normal tension of the ocular muscles.

The three causes would then be, according to the author, Congenital, Hyperemia and Congestion, and Intra-ocular pressure.

The writer does not concur with many in believing the myopic eye of about 3.0 D. the best eye for general use through life. The opinions of some of the German writers on this point are quite pronounced, perhaps, in some cases, because they are themselves myopes.

Lecture VI. treats of the application of the Ophthalmoscope in determining the degree of refraction, and the different modes of examining the eye. This chapter, one of the most difficult in ophthalmology, is rendered comparatively easy by the many schematic illustrations, and the author's painstaking style.

Lecture VII. is devoted to the discussion of muscular asthenopia, the different forms of strabismus, etc. As to the cause of amblyopia in a squinting eye, the writer does not believe it due to a non-use of the retinal elements, and suppression of the image on the retina, but thinks it congenital; the eye, unable to fix its visual axis upon an object, turns inward, and a convergent strabismus follows.

In Lecture VIII. are discussed the different forms of astigmatism, their detection by means of the ophthalmoscope, and their treatment by cylindrical glasses.

Lecture IX. is devoted to Retinoscopy, Pupiloscopy, or the Diagnosis of the Different Forms of Refraction by the Shadow Test. This method renders great service in the examination of illiterate persons, young children, and those with whom the other tests are impossible. The author deals with this subject in the same simple, concise manner which characterizes his whole work, rendering it at once plain and comprehensible.

Lecture X. treats of Presbyopia, and Lecture XI. is devoted to illustrative cases from private and clinical practice. The latter lecture deals with the practical part of Refraction, the clinical history, examination, and treatment of a number of cases are here reproduced.

The lectures, as a whole, are commendable alike to practitioner and specialist, being neither too technical for the former, nor too superficial for the latter. For a more thorough study of Refraction the reader is referred to the works of Donders, Landolt, etc.

This work, from the press of G. P. Putnam's Sons, is in keeping with the excellent work done by this house. Paper, print and illustrations are of the first quality, points which the reader does not care to overlook in these days of hasty and voluminous publications.

